# Pitting the olive seed microbiome

**DOI:** 10.1186/s40793-024-00560-x

**Published:** 2024-03-15

**Authors:** Nuria M. Wentzien, Antonio J. Fernández-González, Antonio Valverde-Corredor, Ana V. Lasa, Pablo J. Villadas, Wisnu Adi Wicaksono, Tomislav Cernava, Gabriele Berg, Manuel Fernández-López, Jesús Mercado-Blanco

**Affiliations:** 1 Departamento de Microbiología del Suelo y la Planta, Estación Experimental del Zaidín, Consejo Superior de Investigaciones Científicas (CSIC), Granada, Spain; 2 Departamento de Protección de Cultivos, Instituto de Agricultura Sostenible, CSIC, Córdoba, Spain; 3 Institute of Environmental Biotechnology, Graz University of Technology, 8010 Graz, Austria; 4 School of Biological Sciences, Faculty of Environmental and Life Sciences, University of Southampton, SO17 1BJ Southampton, UK

**Keywords:** *Cladosporium*, *Malassezia*, *Olea europaea*, Olive genotypes, *Streptomyces*, Vertical transmission

## Abstract

**Background:**

The complex and co-evolved interplay between plants and their microbiota is crucial for the health and fitness of the plant holobiont. However, the microbiota of the seeds is still relatively unexplored and no studies have been conducted with olive trees so far. In this study, we aimed to characterize the bacterial, fungal and archaeal communities present in seeds of ten olive genotypes growing in the same orchard through amplicon sequencing to test whether the olive genotype is a major driver in shaping the seed microbial community, and to identify the origin of the latter. Therefore, we have developed a methodology for obtaining samples from the olive seed’s endosphere under sterile conditions.

**Results:**

A diverse microbiota was uncovered in olive seeds, the plant genotype being an important factor influencing the structure and composition of the microbial communities. The most abundant bacterial phylum was *Actinobacteria*, accounting for an average relative abundance of 41%. At genus level, *Streptomyces* stood out because of its potential influence on community structure. Within the fungal community, *Basidiomycota* and *Ascomycota* were the most abundant phyla, including the genera *Malassezia*, *Cladosporium*, and *Mycosphaerella*. The shared microbiome was composed of four bacterial (*Stenotrophomonas*, *Streptomyces*, *Promicromonospora* and *Acidipropionibacterium*) and three fungal (*Malassezia*, *Cladosporium* and *Mycosphaerella*) genera. Furthermore, a comparison between findings obtained here and earlier results from the root endosphere of the same trees indicated that genera such as *Streptomyces* and *Malassezia* were present in both olive compartments.

**Conclusions:**

This study provides the first insights into the composition of the olive seed microbiota. The highly abundant fungal genus *Malassezia* and the bacterial genus *Streptomyces* reflect a unique signature of the olive seed microbiota. The genotype clearly shaped the composition of the seed’s microbial community, although a shared microbiome was found. We identified genera that may translocate from the roots to the seeds, as they were present in both organs of the same trees. These findings set the stage for future research into potential vertical transmission of olive endophytes and the role of specific microbial taxa in seed germination, development, and seedling survival.

**Supplementary Information:**

The online version contains supplementary material available at 10.1186/s40793-024-00560-x.

## Background

During the last decade, fascination with the plant microbiome has spread rapidly, leading to a growing body of knowledge. This is not only related to its composition and structure, but also led to new discoveries regarding its influence and key contributions to sustaining the plant holobiont’s fitness, development, productivity and resilience toward different stresses [1, 2]. Extensive research has demonstrated that the plant microbiota is actively involved in numerous beneficial processes. These include, but are not limited to, facilitating nutrient uptake by the plant, helping to suppress pathogens or promoting plant growth [2]. However, it is worth noting that certain plant organs, particularly those directly involved in the production of offspring, have comparatively received less attention [3].

An increasing number of studies has provided evidence for the presence of microbes in seeds [4–7]. Some of these microbes have been shown to play a role in crucial processes such as seed germination. For instance, a study with heat-treated seeds of *Lolium rigidum* aimed at reducing the bacterial population, resulted in a pronounced increase in the dormancy period along with a reduction in plant cytokinin levels [8]. Walitang and colleagues [9] isolated bacterial endophytes with promising plant growth promotion activity. Upon inoculation of seeds with these endophytes, both seed germination and growth of seedlings improved substantially. Furthermore, seed endophytes have been shown to determine pathogen resistance in cereal crops. The seed endophyte *Sphingomonas melonis* has been identified as a protective agent against the seed-borne pathogen *Burkholderia plantarii* [10]. Specific seed endophytes that support drought-tolerant wheat varieties to cope with water deficiency have been reported as well [11]. Despite this importance, the assembly of the seed microbiota is not yet fully understood [12].

Seed microbiome assembly is a highly complex process involving a myriad of factors, such as plant breeding, domestication, plant speciation, microbial inheritance from plant to seed and environmental influence [12]. Recent studies showed a contribution of both maternal (ovules and plant endophytes) and paternal (from pollen) microbiota, as well as the influence of the environmental microbiota in the long term [12]. Moreover, other sources can contribute to the composition of the seed microbiota, such as pollinators. Some of these processes lack experimental validation, which explains our limited understanding of the mechanisms regulating these transmission routes or/and on the peculiarities that may occur among plant species [12]. In the specific case of olive (*Olea europaea* L.), several studies are available on the microbial communities present in different plant organs or compartments such as the carposphere [13, 14], flowers [14], phyllosphere [13, 14], root endosphere [15], and the xylem sap [16]. However, almost no experimental evidence has been gathered in terms of the transmission of this microbiota (or specific constituents) to the seeds. Abdelfattah and colleagues [14] documented a striking similarity in the fungal communities found in olive drupes and their source organs (i.e. flowers). Moreover, the transmission of the microbiota from the seed to the seedling is also an emerging research topic and is connected with plant health [17, 18]. Despite the availability of empirical evidence of the influence of the seed microbiota on seed germination and seedling growth [8, 19, 20], much remains to be explored regarding the detailed mechanisms underlying these processes [17]. In addition, microbiome breeding, that is, the modulation of the transmitted microbiome through breeding and the impacts on plant health, with seeds playing a major role in determining microorganisms’ transmission, is a conceptual framework of increasing interest [18, 21].

In order to understand the processes related to seed microbiome assembly and heritability, the first step is to unravel which microorganisms are present in seeds of different crops. Recently, a meta-analysis conducted by Simonin and colleagues [4] has stressed the fact that most of the available studies exploring and unveiling the seed microbiome have been focused on herbaceous plants. In contrast, and despite their economic, social and ecological relevance in a range of agroforestry ecosystems, the seed microbiome of woody plants has been poorly investigated [22–25]. In the case of the olive tree, there is currently no data available related to its seed microbiome [26]. Olive cultivation and the production of olive oil are crucial components of the Mediterranean economy, culture and society [27, 28]. Given the economic significance of olive oil production and the growing concern about the effects that climate change may pose on fruit yield and oil content and quality [29], it is important to unravel the composition of the microbiota present in olive seeds. This knowledge could serve as the foundation for upcoming studies that delve into potential vertical transmission processes of the olive tree microbiota. Moreover, it will be relevant for the assessment of the impact that the seed microbiota has on germination, for breeding programs [30–33], and for targeted isolation of microorganisms advantageous for the health, development, adaptation and resilience of the olive holobiont [26].

In this study, we aimed to address the following objectives: (i) unraveling the composition of the olive seed microbiota using genotypes from different geographical origins and genetic pools, (ii) describing, if any, shared microbiota constituents among different olive genotypes, and iii) examining whether specific constituents of the seed microbiota may originate from the belowground compartment of the tree (i.e. the root endosphere). In addition, we tested the hypothesis that the olive genotype is an important factor in shaping the olive seed microbiota. For this study, a methodology was developed to obtain seed endosphere samples from olive pits under sterile conditions to ensure that microbial DNA originates exclusively from this reproductive organ.

## Methods

### Sample collection

Olive drupes were collected from the World Olive Germplasm Collection (WOGC) (37°51′38.11″N; 4°48′28.61″W; 102 m above sea level) located at the Instituto de Investigación y Formación Agraria y Pesquera (IFAPA, Córdoba, Spain). The sampling was carried out in November 2019. While all trees are grown in the same site, development of the fruit varied to some extent depending on the genotype, ranging from the onset of ripening, veraison or completed fruit ripening [34]. Ten olive genotypes were selected, based on geographical origin and commercial interest as the main criteria of choice (Table 1), eight of them correspond to genotypes used in agriculture (*Olea europaea* L. subsp. *europaea*) and two correspond to wild genotypes (*Olea europaea* L. subsp. *europaea* var. *sylvestris*). The surveyed genotypes are all grown in the same orchard, thereby variability related to agricultural practices, physicochemical properties of the soil, water supply or weather conditions are minimized [15]. Drupes were collected randomly from different branches of two different trees of each genotype, in order to account for possible variability among trees. Eventually, ten healthy drupes from each olive genotype with no visible external damages (i.e. bites, stings or punctures caused by arthropods or birds or any other damage/malformation caused by abiotic factors or pathogens) were selected to be further processed.

**Table 1:** Origin and sampling details of the olive genotypes under study

Genotype	Country of origin	Geographical area	Genetic pool	Number of samples	
				Bacteria	Fungi
Arbequina	Spain	Central MB	Q2	10	10
Barnea	Israel	Central MB	Q2	9	10
Frantoio	Italy	Central MB	Q2	6	8
Jaen (var. *sylvestris*)	Spain	West MB	WW	10	10
Kalinjot	Albania	Eastern MB	Mosaic	6	10
Koroneiki	Greece	Central MB	Q2	10	10
Menorca (var. *sylvestris*)	Spain	West MB	WW	10	8
Picual	Spain	West MB	Q1	10	10
Uslu	Turkey	Eastern MB	Mosaic	8	10
Verde Verdelho	Portugal	West MB	Mosaic	9	10

For each genotype, country of origin, geographical area and classification into genetic pools as defined by Díez and colleagues [35] are shown. The number of samples (seeds) eventually retained per olive genotype after trimming host plant reads and removing samples with less than 500 sequences (see the ‘Illumina data processing’ section) are also displayed, both for the prokaryotic and the eukaryotic datasets. WW: Wild West (*Olea europaea* L. subsp. *europaea* var. *sylvestris*), MB: Mediterranean Basin, Q1: genetic pool 1, Q2: genetic pool 2

### Olive drupe manipulation, endocarp sterilization and seed extraction

In order to avoid seed contamination during the extraction process, the endocarp was meticulously stripped of other tissues and surface sterilized. First, the epicarp and the mesocarp of the fruits were removed using a knife. After that, the endocarp (olive pit) was thoroughly cleaned with a scouring pad to remove any remaining material. Pits fully devoid of fleshy parts were rinsed in water and left to dry completely before further processing. Then, stones were surface sterilized as follows: (i) immersion in 96% ethanol for 1 min, (ii) soaking in diluted commercial bleach (10% v/v) for 1 min, and finally (iii) three rinses in sterile distilled water.

**Fig. 1:**
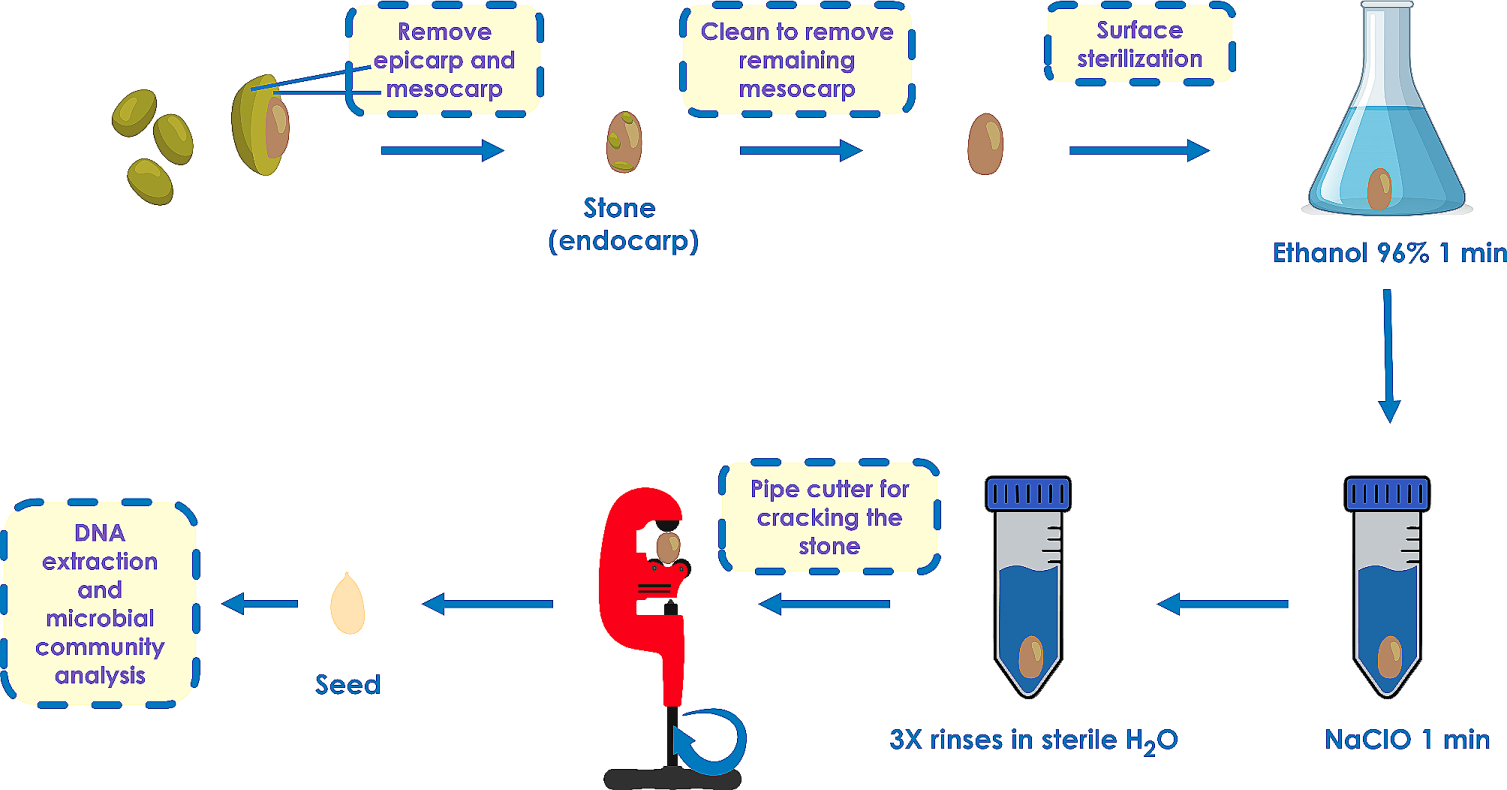
Schematic representation of the procedure implemented to obtain olive seed samples under sterile conditions

Once the endocarp was sterilized, seed samples were obtained by cracking the olive pit using a standard pipe cutter, previously sterilized with 96% ethanol. Eventually, each seed was extracted from the cracked stone using sterile forceps and stored in a microcentrifuge tube. This was chosen as the best method for seed extraction, considering the hardness of the olive stone. Seeds were lyophilized and stored at -80 °C until DNA extraction was conducted. The entire process of olive seed extraction is shown in Fig. 1 and Additional File 1.

### DNA extraction and Illumina sequencing

DNA extraction was performed separately for each individual seed. First, the seed was manually ground under sterile conditions. Afterwards, DNA extraction was done using the Maxwell® RSC PureFood GMO and Authentication Kit for Food, Feed and Seed Samples (Promega Biotech Ibérica S.L., Madrid, Spain) following the manufacturer’s recommendations. DNA yield and quality were checked both by electrophoresis in 0.8% (w/v) agarose gels stained with GelRed and visualized under UV light, and using a Qubit 3.0 fluorometer (Life Technologies, Grand Island, NY, USA).

The DNA was sequenced with the Illumina MiSeq platform in a commercial sequencing service (Instituto de Parasitología y Biomedicina “López Neyra”, CSIC, Granada, Spain) in one run, targeting the V3-V4 hypervariable regions of the *16S rRNA* gene and the ITS2 region, for bacteria and fungi, respectively. The primer pairs used were Pro341F-Pro805R (5’-CCTACGGGNBGCASCAG-3’, 5’-GACTACNVGGGTATCTAATCC-3’) [36, 37] for the *16S rRNA* gene and fITS7-ITS4 (5’-GTGARTCATCGAATCTTTG-3’, 5’-TCCTCCGCTTATTGATATGC-3’) [38, 39] for the ITS2 region. In order to reduce plastid and mitochondrial DNA amplification in the *16S rRNA* gene library, a nested-PCR protocol was implemented, thereby increasing the specificity of the amplification. The first PCR was done with the primer pair 63UF-1115UR (5′-CAGGCCTAACACATGCAAGTC-3′, 5′-AGGGTTGCGCTCGTTG-3′) [40, 41]. Reaction conditions were: 95 °C for 5 min; 25 cycles consisting of 95 °C for 30 s, 52 °C for 30 s and 72 °C for 1:30 min, with a final extension at 72 °C for 5 min. The PCR reaction mixtures contained 12.5 µl of AccuStart™ II PCR ToughMix (Quantabio, Beverly, USA), 1 µl of each primer (10 µM), 8.5 µl of H_2_O and 2 µl of DNA template. The PCR products were purified using Illustra™ MicroSpin™ S-300 h Columns (GE Healthcare Life Sciences, Chicago, Illinois, USA) and sent to the sequencing service, where the second PCR was performed. This second PCR targeted a region within the amplicons generated in the first PCR, corresponding to the sequenced region (with primers Pro341F-Pro805R). Furthermore, PNA PCR clamps were included to further reduce plastid and mitochondrial DNA amplification [37]. The final concentration of PNA PCR clamps was 10 µM. Reaction condicions were: 95 °C for 3 min; 25 cycles consisting of 95 °C for 30 s, 75 °C for 10 s, 55 °C for 30 s and 72 °C for 30 s, with a final extension at 72 °C for 5 min. Both runs were sequenced using a paired-end 2 × 275-bp strategy.

### Illumina data processing

Raw reads were processed following our recently published tutorial available on GitHub (https://nuriamw.github.io/micro4all/) which uses our R package Micro4all [42]. DADA2 was used to infer Amplicon Sequence Variants (ASVs) [43]. For this analysis, we modified some parameters. Specifically, in the quality filtering step, the function *filterAndTrim* was used and the parameter *maxEE* was set to 1 and 2 maximum expected errors for forward and reverse reads for the prokaryotic dataset, according to Figaro tool [44], and to 2 and 5 for the eukaryotic dataset. Merging of forward and reverse reads was done with default parameters. In prokaryotic dataset, reads smaller than 402 and larger than 428 nt were discarded. Finally, classification of bacterial and fungal ASVs was achieved using the *assignTaxonomy* command which implements the RDP naive Bayesian classifier method [45], against the Ribosomal Database Project II, training set v.18 [46] and the UNITE v.7.2 dynamic database [47]. ASVs corresponding to unknown sequences and host DNA (i.e. those that were classified as mitochondria and chloroplasts) were removed. Eukaryotic ASVs that were not classified as fungi at the kingdom level were also removed. Subsequently, ASVs accounting for less than 0.005% of the total sequences were removed according to Bokulich and co-workers [48]. Finally, those samples that ended up with less than 500 sequences were not considered for further analysis.

### Microbial diversity and differential abundance analyses

Alpha diversity indices (i.e. Observed ASV [richness], Shannon, Inverse of Simpson and Pielou’s evenness), rarefaction curves and beta diversity (determined by PCoA based on Bray-Curtis dissimilarities) were computed as previously described by Fernández-González and co-workers [49] using *R* statistics. Normalization was performed using rarefaction for alpha diversity and the “trimmed means of M” (TMM) method from the package *edgeR* for beta diversity [50]. Statistical tests included Kruskal-Wallis (to compare α-diversity indices), Mann-Whitney-Wilcoxon *post-hoc* test, Permutational Multivariate Analysis of Variance (PERMANOVA), Multivariate homogeneity of groups dispersions (BETADISPER) for β-diversity analyses and pairwise Adonis. Analysis of Similarity (ANOSIM) was carried out to confirm whether differences between groups were greater than within groups (function *anosim* from R package vegan [51]). Moreover, differences in taxonomical abundances were assessed with ANCOM-BC [52]. In order to study the relation between the genetic distance of olive genotypes and their seed microbial communities, genotypes were grouped according to the corresponding genetic pool, as previously described by Díez and colleagues [35]. Based on that grouping, α-diversity, β-diversity and ANCOM-BC analyses were performed as described above.

### Shared microbiome

The shared microbiome among genotypes was assessed using the *R* package microbiome [53]. The premises taken into account were: (i) only genera present in at least 50% of the replicates of each genotype were considered for further analysis, and (ii) those genera present in at least 6 out of 10 genotypes.

### Identification of ASVs present in both the seed and the root endosphere of olive trees

In order to evaluate whether specific constituents of the seed microbiota may originate from the roots, the genera common to both olive seeds and the root endosphere were analysed using data from a previous study [15]. This approach could only be implemented for those genotypes examined herein and in the above-mentioned study (i.e. cultivars [cvs.] Arbequina, Picual, Koroneiki, Uslu and Frantoio), considering that sampled trees were exactly the same ones in both studies. It is worth mentioning that DNA samples of the root endosphere of cvs. ‘Picual’ and ‘Frantoio’ could not be included in the *16S rRNA* gene analysis of our previous work [15] because they did not reach a sufficient number of sequences after quality check. Thus, these samples were re-sequenced including PNA clamps as described above in detail except for the nested-PCR protocol. Finally, the root endosphere samples were subjected to ASV inference following the same procedure as for the seed samples. Those genera present in at least 50% of the replicates here described were considered for analysis and compared to the previously-published data (considering only those genera present in all root endosphere samples), both for bacteria and fungi. In addition, we investigated which bacterial ASVs were exclusively present in olive seeds when compared with root endosphere samples. For this, we only considered ASVs with a mean relative abundance > 1% in olive seeds (average of the relative abundance among all samples) and with a prevalence > 19% (i.e. ASVs with a relative abundance greater than 0 in at least 19% of the samples). For this analysis, all genotypes were included.

### Archaeal quantification

We also aimed to unveil a potentially occuring archaeal community in the olive seeds by sequencing archaea-specific *16S rRNA* gene fragments, as described by Taffner and colleagues [54]. However, the nested-PCR step yielded no amplification. In order to confirm the absence or very low abundance of archaea, we aimed to quantify the copy number of *16S rRNA* gene with primers specific to this domain. Real-time PCR experiments were conducted for that purpose. For each olive genotype, two samples from two different trees were used for quantification. Primers 344aF (5’-ACGGGGYGCAGCAGGCGCGA-3’) and 517uR (5’-GWATTACCGCGGCKGCTG-3’) were implemented [55]. The concentration of template DNA ranged from 0.06 to 0.4 ng. Reaction conditions were: 95 °C for 5 min; 40 cycles consisting of 95 °C for 15 s, 62 °C for 30 s and 72 °C for 30 s. Melting curves were obtained as well by increasing the temperature from 62 to 95 °C. For the construction of calibration curves, the *16S rRNA* gene from template DNA of an environmental sample highly enriched in *Archaea* was cloned into pGEM-T Easy Vector System (Promega Biotech Ibérica S.L, Madrid) using the same primers as in the quantification and following the manufacturer’s recommendations. The cloning product was checked with NotI digestion and PCR with 344aF/517uR primers followed by agarose gel electrophoresis. Mixes were prepared using the TB Green Premix Ex Taq II (Tli RNase H Plus) (Takara Bio Europe SAS, Saint-Germain-en-Laye, France) and reactions were carried out with QuantStudio™ 3 Real-Time PCR System (Applied Biosystems™, Alcobendas, Spain). The qPCR reaction mixtures contained 5 µl of TB Green Premix, 0.5 µl of each primer (5 µM), 3 µl of H2O and 1 µl of DNA template. Results were analyzed with QuantStudio Design & Analysis Software v1.5.2 (Applied Biosystems™, Alcobendas, Spain).

## Results

### General characteristics of sequencing datasets

The total number of raw reads obtained for bacterial and fungal datasets were 7,370,663 and 7,312,666, respectively. The number of sequences after quality filtering, ASVs inferring and removing chimera and host plant reads, was 327,237 for bacterial and 4,781,620 for fungal communities. After removing samples with less than 500 reads, a minimum of 513 and a maximum of 41,633 sequences per sample were retained from the prokaryotic dataset; 9,643 and 279,514 sequences from the fungal dataset. The final number of ASVs retained for further analyses was 1,206 (bacterial) and 1,084 (fungal). The final number of replicates (seeds) kept for further analyses is detailed in Table 1.

### Genotype drives microbial community diversity and structure

**Fig. 2:**
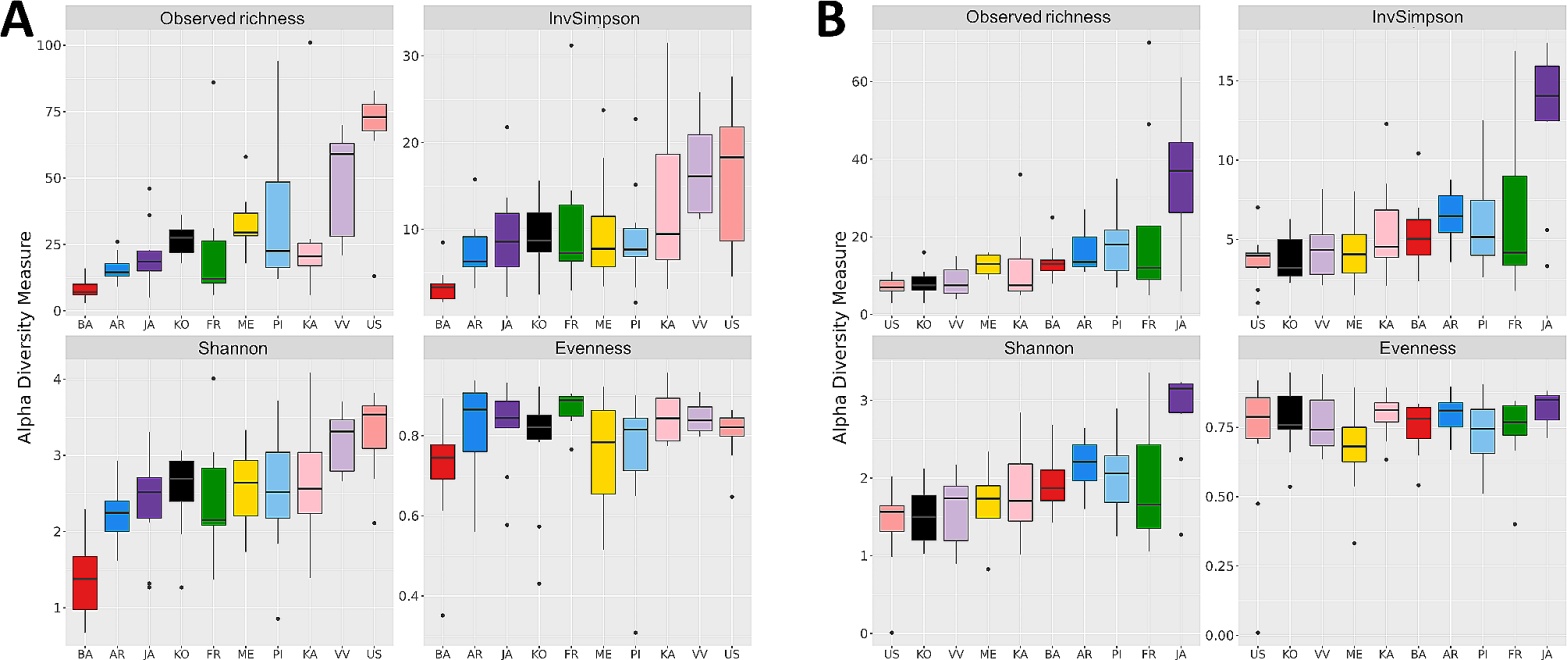
Bacterial (**A**) and Fungal (**B**) α-diversity indices of each olive genotype. For each panel, five summary statistics (the median, two hinges and two whiskers) and outlying points are shown. AR: Arbequina, BA: Barnea, FR: Frantoio, JA: Jaén, KA: Kalinjot, KO: Koroneiki, ME: Menorca, PI: Picual, US: Uslu, VV: Verde Verdelho

**Fig. 3:**
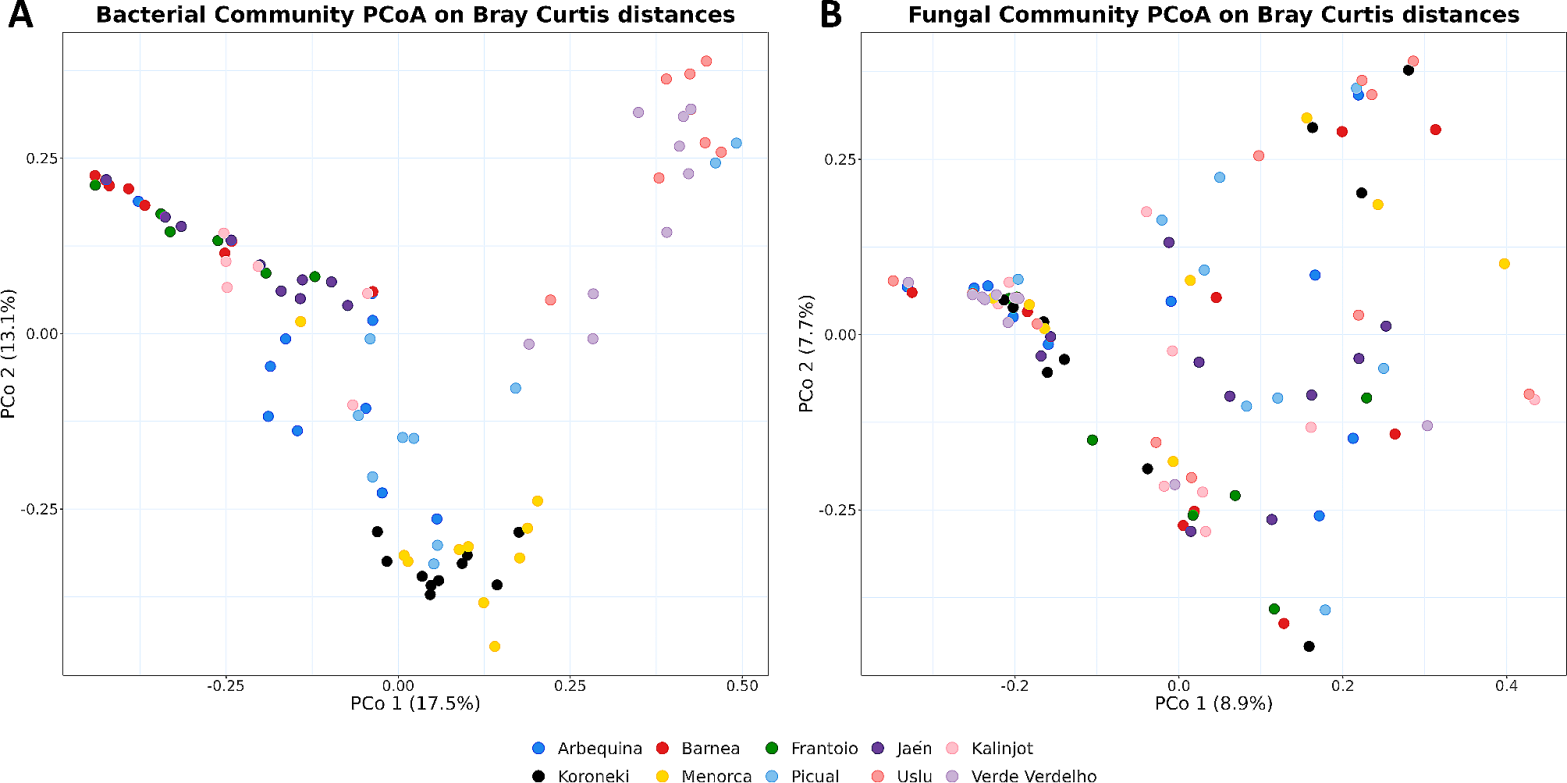
Principal coordinates analyses of the bacterial (**A**) and fungal (**B**) communities. Ordination plots were calculated using Bray-Curtis dissimilarities for each genotype under study

For both bacterial and fungal communities, statistically significant differences were found for Shannon index, Inverse of Simpson and Observed Richness (*p* < 0.05, Kruskal-Wallis test) when comparing olive genotypes (Table S1, Additional File 2). Interestingly, cv. ‘Barnea’ was the genotype with the lowest bacterial diversity and richness, while cvs. ‘Verde Verdelho’ and ‘Uslu’ showed the highest values (Fig. 2A). The opposite pattern was observed for the fungal community, cv. ‘Uslu’ being the genotype with the lowest fungal richness and diversity (Fig. 2B and Table T1, Additional file 3). In line with these findings, community structure was also shown to be determined by the genotype, as indicated by β-diversity analysis. Specifically, PERMANOVA resulted in statistically significant differences for both datasets (Table S2, Additional File 2) with high variability explained by the genotype (R^2^ = 0.34 and R^2^ = 0.11 for bacterial and fungal communities, respectively). Although the beta dispersion was significantly different among genotypes for the prokaryotic dataset (*p* = 0.003 according to a multivariate test of homogeneity of group dispersions), ANOSIM test (*p* = 0.001, Table S2, Additional file 2) and PCoA plots confirmed a separation among genotypes (Fig. 3A and B). Moreover, three clear groups were distinguished in the bacterial community (i.e. genotypes with no statistically significant differences among them), namely genotypes ‘Frantoio’-‘Jaen’-‘Kalinjot’, ‘Uslu’-‘Verde Verdelho’ and ‘Koroneiki’-‘Menorca’ (adjusted *p* > 0.05, *pairwiseAdonis* test, Table T4, Additional file 3). For the fungal community, in contrast, no statistically significant differences were found between pairs when correcting *p-*values (Table T3, Additional File 3).

### Genetic pool influences seed bacterial community

According to Díez and colleagues [35], olive genotypes can be grouped into different genetic pools which correlate with their geographical origin. We investigated whether these groups could explain some of the variations in the seed microbial community observed among genotypes. In our present study, representatives of four genetic pools, namely Q1, Q2, Mosaic and West Wild (WW) were included (Table 1). In this regard, β-diversity analysis showed that genetic pools explained 12% of the variation among genotypes for the bacterial community (PERMANOVA test, *p*-value = 0.001, R^2^ = 0.12), with no differences in the beta dispersion (PERMDISP2 test, *p* = 0.26; Table S2 and Figure S1, Additional File 2). Moreover, differences were found between all of the groups, as shown by a *pairwiseAdonis* test (adjusted *p* < 0.05, Table T5, Additional file 3). The same analysis was conducted for the fungal community, but no statistically significant difference was found in this case (Table S2, Additional file 2).

### First description of the olive seed microbiome: composition and shared microbiome

**Fig. 4:**
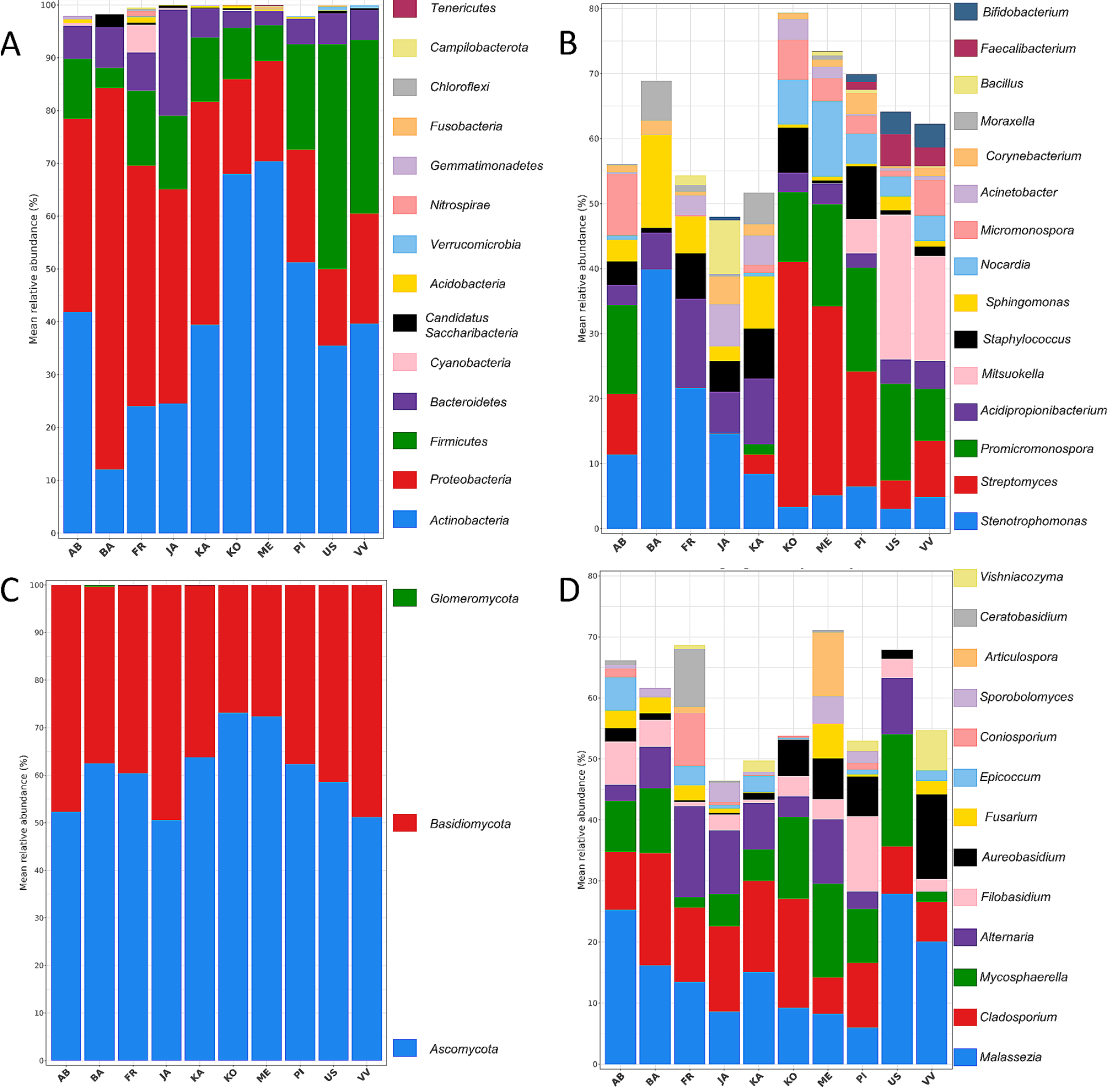
Bacterial (**A**, **B**) and fungal (**C**, **D**) taxonomic profiles from seeds of different olive tree genotypes. Microbial composition is shown at phylum (**A**, **C**) and genus level (**B**, **D**). The 15 most abundant taxa are presented. AR: Arbequina, BA: Barnea, FR: Frantoio, JA: Jaén, KA: Kalinjot, KO: Koroneiki, ME: Menorca, PI: Picual, US: Uslu, VV: Verde Verdelho

The bacterial taxonomic profiles at phylum level were dominated by *Actinobacteria*, *Proteobacteria*, *Firmicutes* and *Bacteroidetes* which accounted for at least 91% of the sequences in all olive genotpyes (Fig. 4A). At genus level, differences among genotypes were more pronounced. The most abundant genera were *Stenotrophomonas*, *Streptomyces*, *Promicromonospora* and *Acidipropionibacterium* (Fig. 4B), although their abundances varied depending on the genotype. Moreover, these genera were part of the shared bacterial communities within olive seeds, i.e. genera with a sample prevalence of more than 50% and present in at least 6 out of 10 genotypes analysed. Regarding *Streptomyces*, some genotypes could be grouped according to the relative abundance of this genus, i.e. genotypes within the same group exhibited similar levels of *Streptomyces*. This observation was supported by ANCOM-BC, which indicated no statistically significant differences within groups (adjusted *p* > 0.05, Additional file 4). Interestingly, these groups were the same as shown by β-diversity analysis, genotypes ‘Frantoio’-‘Jaén’-‘Kalinjot’ (i.e. *Streptomyces* was almost absent), genotypes ‘Koroneiki’-‘Menorca’ (i.e. *Streptomyces* was the main genus) and cvs. ‘Uslu’-‘Verde Verdelho’ (i.e. showing an intermediate profile).

As for the fungal community, the most abundant phyla were *Basidiomycota* and *Ascomycota* (Fig. 4C). In fact, these two phyla accounted for 100% of the sequences in most genotypes. Remarkably, seeds of three genotypes (‘Barnea’, ‘Kalinjot’ and ‘Frantoio’) also harboured members of the genus *Rhizophagus* (phylum *Glomeromycota*), which accounted for 0.33, 0.04 and 0.06% of the sequences, respectively. Regarding the composition at genus level, the communities were mainly dominated by *Malassezia*, *Cladosporium* and *Mycosphaerella* (Fig. 4D). These genera also formed part of the shared seed microbiome of these genotypes. *Malassezia* was present in all genotypes in more than 50% of the samples.

As for the quantification of *Archaea*, real-time PCR experiments did not yield enough differentiation between the tested samples and the basal amplification of the negative control. Thus, under the experimental conditions used in this study, no *Archaea* were detected in olive seeds.

### Identification of ASVs present in both the seed and the root endosphere of olive trees

**Fig. 5:**
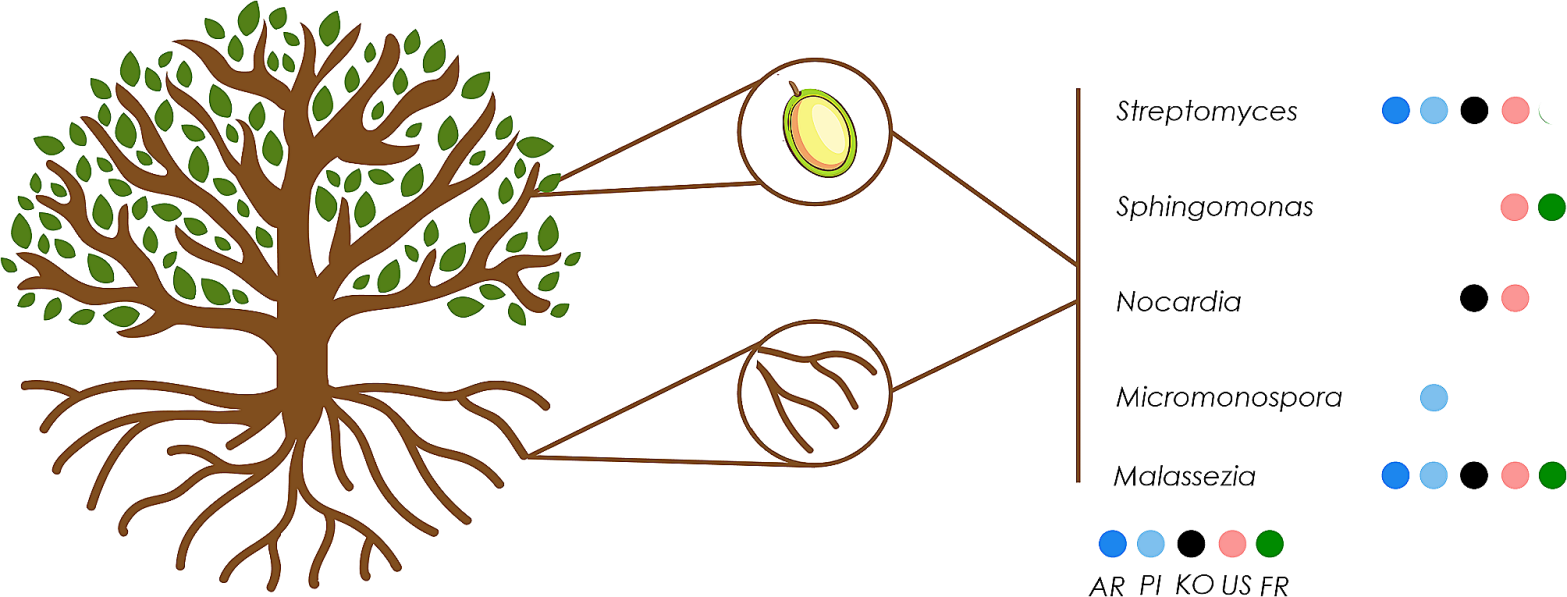
Genera present in seeds and the root endosphere of the same genotypes. Commonalities found in five olive genotypes are shown (see main text for details). AR: Arbequina, FR: Frantoio, KO: Koroneiki, PI: Picual, US: Uslu

In order to examine whether specific components of the root endosphere microbiome are also present in the seed microbiome, a scenario suggestive of acropetal movement of endophytes from roots to seeds within the same olive tree, the shared microbiome among samples of these organs was assessed. This was performed both at bacterial and fungal community level for the five genotypes in common in both studies (i.e. ‘Arbequina’, ‘Picual’, ‘Koroneiki’, ‘Uslu’ and ‘Frantoio’). The bacterial community found in both seeds and root endosphere was mainly composed of *Actinobacteria* members, i.e. *Streptomyces*, *Micromonospora* and *Nocardia*. A member of the phylum *Proteobacteria* (*Sphingomonas*) was also detected. *Streptomyces* was found in all genotypes, with the exception of ‘Frantoio’. Regarding the fungal community, *Malassezia* members were present in both compartments and in all genotypes under study (Fig. 5).

Regarding the microbiome fraction specific to olive seeds, a total of five ASVs were present in the olive seeds, but not detected in the root endosphere, with a relative abundance > 1% and a prevalence > 19%. At the genus level, these ASVs were classified as *Stenotrophomonas*, *Micromonospora*, *Mitsuokella* (two ASVs) and *Streptomyces*, with a mean relative abundance of 11.40, 2.69, 1.99, 1.25 and 1.15%, respectively.

## Discussion

We described the olive seed microbiota for the first time and found that it is highly unique in comparison with other plant species. However, some general similarities were found. Firstly, the microbial community is dominated by four bacterial phyla: *Actinobacteria, Proteobacteria*, *Firmicutes* and *Bacteroidetes* and two fungal phyla: *Basidiomycota* and *Ascomycota*. This outcome is consistent with a recent meta-analysis of seed samples from 50 distinct plant species [4]. At the genus level, and in accordance with the seed composition of other plant species [4], *Cladosporium* was highly abundant in the fungal community of olive seeds and appeared as a member of the shared mycobiome of the genotypes analysed. This outcome confirms previous studies focused on endophytes originating from different olive organs or compartments, such as leaf, fruit, flower, twig and the root endosphere [14, 15, 42, 56, 57]. Moreover, members of this genus have been associated with potential biocontrol activities against olive phytopathogens [58, 59]. These results encourage further efforts aimed to isolate and characterize members of this genus from different olive tissues/organs, including seeds.

In addition to the aforementioned similarities to other plant species, distinctive features of the seed microbiota of olive trees were revealed in our study. First of all, *Actinobacteria* was the most abundant bacterial phylum, while most of the plant seeds so far studied show a predominance of *Proteobacteria*. *Actinobacteria* was predominantly found also in the root endosphere of the same trees in an earlier study [15], and also in cv. ‘Picual’ grown in a commercial orchard located at a distant geographical site and subjected to different agronomic management [42]. Actinobacterial members are known for their ability to feed on complex sugars [60], which are the primary carbohydrates supply in sink organs such as roots and seeds [61, 62]. However, there is limited knowledge regarding the specific conditions that promote the growth of *Actinobacteria* in certain parts of this tree species, as well as the significance of their role in shaping the overall microbial community and the impact on the olive holobiont’s health [15, 63]. In line with this result, the shared fraction of the olive seed’s bacterial community is primarily composed of actinobacterial members. Specifically, *Streptomyces* seems to be a key member of the microbial community. This is reinforced by the fact that certain olive genotypes could be grouped according to the relative abundance of this genus, and clustering consistent with the pattern observed when analyzing the community structure using Bray-Curtis dissimilarities. *Streptomyces* was previously found as one of the most abundant genera in the root endosphere of the same trees here analysed [15] and in cv. ‘Picual’ trees grown at a distant site [42]. Members of *Streptomyces* are widely known for their beneficial role in supporting plant health, with very few species causing plant diseases [64, 65]. Although no studies have been conducted on the role of *Streptomyces* on seed germination, some *Streptomyces* isolates are known to produce auxin [64, 65]. This plant hormone has been described to participate in the regulation of seed germination, promoting seed dormancy [66–68]. Moreover, auxin is known to positively regulate seed and fruit development, being produced in the endosperm [69, 70]. In this sense, *Streptomyces* present in olive seeds could play a role in fruit growth as well as seed germination. Other members of the phylum *Actinobacteria* present in the shared seed microbiome were *Promicromonospora* and *Acidipropionibacterium*. Interestingly, a member of *Promicromonospora* producing gibberellins, a plant hormone that regulates plant’s growth, seed germination, flowering and fruit production, among others, has been earlier described [71]. Regarding *Acidipropionibacterium* spp., they have been mainly studied in lactic products as biopreservatives because of the production of propionic acid, which acts as an antifungal agent [72]. Finally, it is worth noting that genera with interesting functions for human health were identified in the olive seed microbiota as well, such as *Faecalibacterium*. For instance *Faecalibacterium prausnitzii* has been found to account for up to 5% of the fecal human microflora and be depleted in patiens with microbial dysbiosis, thus being a very promising probiotic [73, 74].

Regarding the fungal community composition, *Malassezia* was the most abundant and prevalent fungal genus, being a highly distinctive feature of the olive seed microbiota [4]. Although research is mainly focused on its effects on human health, *Malassezia* members have been detected in different ecosystems and niches, including marine sediments and roots of orchids [75]. Intriguingly, all *Malassezia* species except *M. pachydermatis* depend on external lipid supplies for survival, as their fatty acids synthesis metabolic pathway is incomplete [76]. Since the endosperm of olive seeds harbours a high content of fatty acids [77], the growth of *Malassezia* could be favoured in this organ. Additionally, the presence of *Glomeromycota* in olive seeds is another intriguing characteristic. While transmission of arbuscular mycorrhiza fungi to plant seeds seems to be rare [4], the presence of *Glomeromycota* has been demonstrated in three olive genotypes analyzed in our study (cvs. ‘Frantoio’, ‘Barnea’, and ‘Kalinjot’) although at very low relative abundance level. A low abundance of *Glomeromycota* has also been reported in seeds from other woody plants, such as oak (*Quercus robur*) [25]. The early colonization of mycorrhizal fungi can be considered an important factor in the successful establishment and growth of seedlings [78]. Our finding could be of relevance for olive breeding programs, as seed selection based on the presence of *Glomeromycota* may increase seedling growth and survival rates, especially in the case of olive which has a protracted juvenile phase. Overall, efforts to isolate and characterize representatives of these genera would be crucial not only for deciphering their role within the seed and in shaping the microbial community structure in this organ, but also for identifying potential biotechnological applications in agriculture from the early stages of the plant development.

According to our findings, the genotype strongly determined both the bacterial and fungal communities of olive seeds. Following this, clustering genotypes based on the genetic pools outlined by Díez and colleagues [35] also explained a large level of variability, but only for the bacterial community (12%). These genetic pools were based on the sequencing of nuclear simple-sequence repeat (SSR) markers. Thus, the variability explained by this clustering is closely related to the genetic variability among genotypes. In this sense, the importance of the genotype as an influencing factor on the composition of the microbial community has been stressed in different olive compartments/organs. For instance, Müller and colleagues [55] found that the genotype had a stronger impact on bacterial and archaeal communities of olive leaves when compared with soil and climate conditions. These results were confirmed by Malacrinò and colleagues [13] for olive leaves, soil and fruits. The importance of the genotype has also been demonstrated for belowground compartments [15] and in the xylem sap [79].

Other interesting details worthy of being discussed are the sources from which the olive seed microbiome originates and what could be their fate (or that of some of its constituents) concerning potential vertical transmission events. These sources include endophytes inhabiting plant compartments such as roots, flowers or the xylem sap, as well as microorganisms carried by pollinators and gametophytes, or present in the surrounding environment [12]. Regarding the olive tree, several studies have focussed on the description of microbial communities found in source organs, such as the carposphere, flowers, phyllosphere and xylem sap [13, 14]. In this context, pollen could play a role in transmitting microorganisms to the pistil and, subsequently, to the seed. Nevertheless, there is a notable absence of experimental evidence for this process in both olive trees and other plant species [12]. Specifically, for olive trees, the microbial composition of pollen has not yet been documented. Since most olive genotypes exhibit self-incompatibility [80] it would be important in future research efforts to consider how this fact might influence the microbial diversity present in olive seeds [12].

Our results provide the first approximation to identify one of the potential sources from which the olive seed microbiome may originate, or at least part of it. Indeed, we have been able to determine a number of shared genera present in the root endosphere and the seeds produced in the same tree. This shared microbiome mainly included *Actinobacteria* members (*Streptomyces*, *Nocardia* and *Micromonospora*) and only one genus from *Proteobacteria* (*Sphingomonas*). Again, the importance of *Streptomyces* was highlighted, not only as a very abundant and prevalent genus in seeds but also in the root endosphere. Interestingly enough, both *Nocardia* and *Micromonospora* strains have shown potential to promote plant growth by various mechanisms [81–83]. In addition, many *Nocardia* species show antibiotic production, which could play a role in seed protection [83]. It is worth mentioning that *Sphingomonas* was present in both seeds and the root endosphere of genotypes qualified as tolerant to *Verticillium dahliae* (i.e. ‘Frantoio’ and ‘Uslu’) [84]; this may suggest a role of this bacterium to confront this relevant pathogen affecting olive cultivation. Our previous study has shown the increased relative abundance of this genus in the root endosphere of cv. ‘Frantoio’ plants in contrast to a genotype susceptible to *V. dahliae*, namely cv. ‘Picual’ [49]. Furthermore, *Sphingomonas* members are also known for its ability to degrade pollutants and confer protection against pathogens [10, 85]. While experimental evidence is still needed to prove the migration of some beneficial microorganisms from olive roots to the seeds, it was already proven that microorganisms can migrate from the root to the leaves through the vascular system [86, 87]. Moreover, the movement from seeds to roots has been previously reported [28, 88].

In contrast, some ASVs were exclusively found in the seeds. The most abundant “olive seed-specific” ASV belonged to *Stenotrophomonas*, a bacterial genus well-known for plant growth promotion and biocontrol activities displayed by some of its members [89]. Other ASVs within this group belonged to genera also characterized by showing beneficial functions for the plant, such as *Micromonospora* [81]. These results indicate that some beneficial microorganisms present in olive seeds may also originate from sources other than belowground compartments, as they were only detected in the olive seed samples, according to our data.

Overall, our findings constitute the starting point for further insights on the origin of the diverse components of the olive seed microbiome here identified, on the possibility that this microbiome (or part of it) can be inherited to the offspring, and on its potential role in seed germination and favoring growth and stress tolerance at early stages of the seedling development.

## Conclusions

The present study unveiled, for the first time, the composition of the olive seed microbiota. This microbial community exhibits distinctive features compared to other plant species. *Malassezia* was identified as the most abundant and prevalent fungal genus in olive seeds, in contrast to what has been so far reported for other plant species. Notably, certain taxa that showed a high relative abundance and prevalence in the seeds have also been extensively documented in other olive tree organs. This apparent systemic colonization of the host underscores the significance of these taxa for the fitness, development and health of the olive holobiont, and suggests that they could be favorably “recruited” by the plant. Moreover, we identified *Streptomyces* as a potential contributor to shaping the microbiota of olive seeds. This study also sheds light on the substantial influence that the olive genotype exerts on both bacterial and fungal communities of the seeds, in line with previous studies focused on other organs of this tree species. Interestingly, and regardless of the genetic pool or the geographical origin from which the olive genotypes here analyzed originated, a shared microbiome was identified. This common microbial community found among genotypes included genera such as *Streptomyces* and *Malassezia*. Besides, occurence of *Streptomyces* and *Malassezia* in the root endosphere and seeds constitutes supporting evidence of a likely systemic colonization of the olive holobiont by members of these taxa, stressing their relevance for the olive holobiont. On the other hand, their presence at both the belowground and aboveground compartments sheds light into a potential migration route, suggesting the possibility that these bacteria and fungi may migrate from the roots to the seeds in olive trees. Their occurrence in the seeds may also indicate these microorganisms could be vertically transmitted. Future research efforts should be focussed on the identification, isolation and characterization of keystone microorganisms of the olive seed microbiota. By doing so, their roles in the processes mentioned above will be better understood. In addition, their agrobiotechnological potential as biomarkers in olive breeding programs and as contributors to tolerance of olive genotypes against a/biotic stressors affecting their cultivation could be assessed.

### Electronic supplementary material

Below is the link to the electronic supplementary material.

Supplementary Material 1

Supplementary Material 2

Supplementary Material 3

Supplementary Material 4

## Data Availability

The datasets generated during the current study are available in the National Center for Biotechnology Information Sequence Read Archive (NCBI SRA) repository, under the BioProject ID PRJNA1017437 (https://www.ncbi.nlm.nih.gov/bioproject/PRJNA1017437). The dataset of the root endosphere samples is included under the BioProject PRJNA498945.
