# Supplementary material for: Pitting the olive seed microbiome
Source: Environ Microbiome. 2024 Mar 15;19:17. doi: 10.1186/s40793-024-00560-x (PMC10943921; doi:10.1186/s40793-024-00560-x)
Supplement: Supplementary file 2 — Supplementary Material 2 [file 40793_2024_560_MOESM2_ESM.pdf]

Table S1. Statistical comparison of alpha diversity indices based on Kruskal-Wallis tests among genotypes.

	Observed OTUs		Inverse of Simpson		Shannon		Evenness	
Comparison	Bacteria	Fungi	Bacteria	Fungi	Bacteria	Fungi	Bacteria	Fungi
Genotype	***$1.84 \cdot 10^{-6}$***	***$6.35 \cdot 10^{-6}$***	***$6.75 \cdot 10^{-5}$***	***0.001***	***$9.15 \cdot 10^{-4}$***	***$2.75 \cdot 10^{-4}$***	0.20	0.48

Numbers in boldface and italics indicate significant *p* values (<0.05).

Table S2. Statistical comparison of beta diversity indices based on Bray-Curtis dissimilarities

	PERMANOVA		R^2		BETADISPERSION		ANOSIM	
Comparison	Bacteria	Fungi	Bacteria	Fungi	Bacteria	Fungi	Bacteria	Fungi
Genotypes	***0.001***	***0.003***	0.34	0.11	***0.003***	0.74	***0.001***	***0.001***
Genetic Pool	***0.001***	0.241	0.12	0.03	0.26	0.169	***0.001***	0.84

Numbers in boldface and italics indicate significant *p* values (< 0.05) according to PERMANOVA and BETADISPER tests among genotypes and genetic pools.

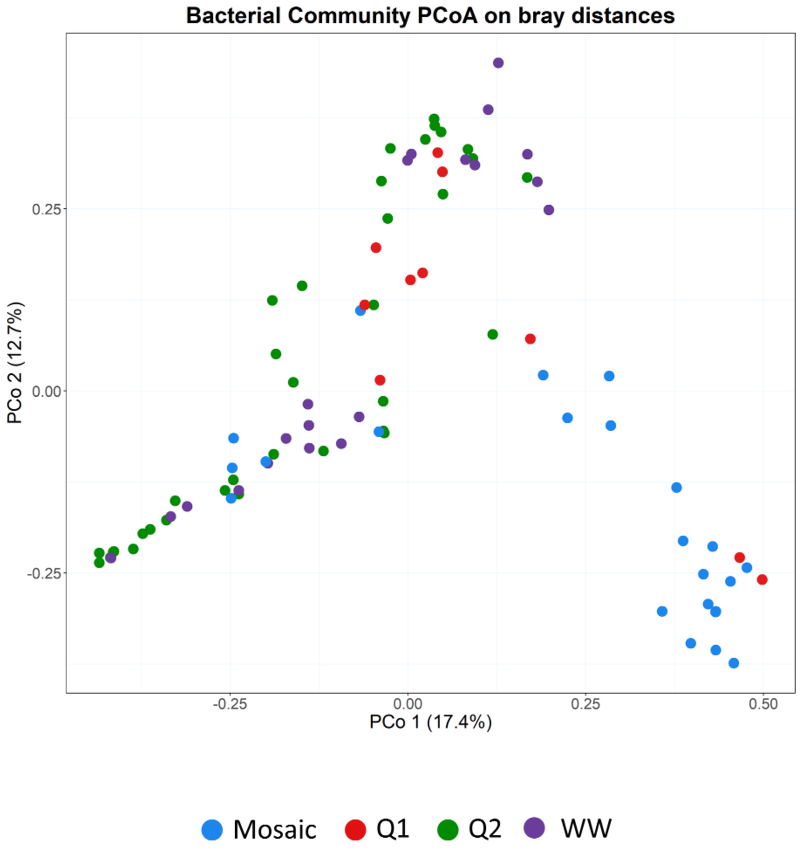

Figure S1. Principal coordinates analyses of the bacterial community. Ordination plot was calculated using Bray-Curtis dissimilarities for genetic cluster under study.
